# Upper Tract Urothelial Carcinoma: An Update on Current Diagnostic Modalities and Emerging Biomarkers

**DOI:** 10.3390/jpm16040220

**Published:** 2026-04-16

**Authors:** Konstantinos Kapriniotis, Ioannis Loufopoulos, Mohammad U. Sharif, Ioannis Manolitsis, Lazaros Tzelves, Amy Nagle, James S. A. Green

**Affiliations:** 1 Department of Urology, Whipps Cross University Hospital, London E11 1NR, UK; 2 Department of Urology, Royal Free Hospital, London NW3 2QG, UK; 3 Department of Urology, Aberdeen Royal Infirmary, Aberdeen AB25 2ZN, UK; giannismanolit@gmail.com; 4 2nd Department of Urology, National and Kapodistrian University of Athens, 151 26 Athens, Greece; lazarostzelves@gmail.com

**Keywords:** urothelial carcinoma, upper tract, diagnosis, prognosis, biomarkers

## Abstract

**Introduction:** Upper tract urothelial carcinoma is a rare disease with variable prognosis depending on the stage and grade at diagnosis. Current modalities are far from perfect in diagnosis and risk stratification. In this setting, there is an urgent need for diagnostic and prognostic biomarkers to overcome these limitations. **Methods:** We carried out a narrative review of the literature searching for research articles on diagnostic and prognostic biomarkers for upper tract urothelial carcinoma (UC) and underlined the limitations of current diagnostic modalities. **Results:** CT urogram (CTU) is the imaging modality of choice in suspected upper tract UC with sensitivity and specificity exceeding 90% but with limitations in smaller lesions. Urine cytology has an excellent specificity for high-grade UC but is limited by low sensitivity leading to a high number of diagnostic ureteroscopies with significant associated risks. Adjuncts such as Fluorescence In Situ Hybridization (FISH) technology and urine DNA methylation markers have shown promising results but need further validation in large cohorts of upper tract UC. Finally, circulation tumour DNA (ctDNA) is a novel approach with great potential in risk stratification and monitoring of minimal residual disease post radical surgery; however, larger prospective studies are required to validate its role similarly to the recent bladder UC trials. **Conclusions:** There is an urgent need for non-invasive biomarkers that can reliably replace diagnostic ureteroscopies, identify high-risk/invasive disease and select patients for radical surgery or kidney sparing procedures.

## 1. Introduction

### 1.1. Epidemiology and Treatment Options of UTUC

Upper tract urothelial cancer (UTUC) is a relatively rare disease with an estimated annual incidence of 2–4 cases per 100.000 people with recent reports suggesting an increasing trend. It accounts for less than 10% of all urothelial carcinomas (UC) and, similarly to bladder cancer, is more common in male patients with an incidence ratio of 2:1 [1,2]. Approximately two thirds of the UTUC are located in the pelvicalyceal system, whereas multifocal upper tract lesions or concomitant carcinoma in situ (CIS) have been reported in over 20% of the cases in large series. Similarly, synchronous bladder cancer has been reported in approximately 15–20% of UTUC cases [3]. Comparing to bladder UC, a significantly higher proportion of patients have high-grade disease and over half of the cases have muscle invasive or locally advanced disease at diagnosis [4]. Although UTUC shares certain characteristics with bladder UC, it has distinct features including stronger association with specific genetic and epigenetic changes such as microsatellite instability (MSI), risk factors such as exposure to aristolochic acid and familial syndromes, most notably the Lynch syndrome [5].

The most common presenting symptoms of UTUC include haematuria and flank pain, whereas generalised constitutional symptoms such as weight loss and malaise are typically associated with advanced disease [6]. Tumour stage and grade are critical for risk stratification, choice of surgical approach and the strongest prognostic factors for disease recurrence and overall survival (OS) after radical treatment. For instance, in large retrospective series, non-invasive disease is associated with 5-year survival rates exceeding 90%, whereas locally advanced disease with rates below 40–50% [7].

The European Association of Urology (EAU) and American Urological Association (AUA) guidelines recommend radical nephroureterectomy as the treatment of choice for high-risk disease, whereas kidney sparing surgery, such as ureteroscopic ablation or less commonly segmental ureterectomy, is recommended in low-risk disease [8,9]. However, current imaging modalities and ureteroscopic assessment with biopsy have significant limitations in assessing the grade and stage of the disease and therefore inform on the appropriate treatment strategy. Therefore, a significant proportion of patients are at risk of under- or overtreatment based on the conventional diagnostic modalities. In addition, decisions regarding performing lymph node dissection at the time of radical surgery or offering neoadjuvant chemotherapy largely depend on tumour stage and lymph node status, which make appropriate preoperative staging and risk stratification critical. Several risk factors acting as surrogates of disease aggressiveness have been described and incorporated in prognostic models including tumour grade, size, uni- or multifocality and appearance on cross-sectional imaging [10].

### 1.2. Molecular Differences Between UTUC and Bladder UC

Although bladder UC and UTUC share histological similarities as tumours originating from the same urothelial lining, they are molecularly different entities in terms of genomic architecture, mutational processes, and tumour microenvironment. The most consistent finding across studies is that FGFR3 alterations, including both activating mutations and gene fusions, are significantly increased in UTUC (occurring in 27–36% of cases) compared to bladder UC (14–22%), with the highest frequency observed in renal pelvis tumours (28%), followed by ureteral tumours (22%) and bladder primaries (18%), suggesting an anatomic gradient of FGFR3 dependency along the urinary tract [11,12]. UTUC is additionally enriched for mutations in HRAS, KMT2D, KRAS, CDKN2B, and MYC, suggesting that upper tract tumours rely more on receptor tyrosine kinase and RAS pathway signalling for oncogenic transformation and maintenance [5,13]. In contrast, bladder UC shows a different pattern of genomic disruption, with enrichment for TP53 mutations (46–58% vs. 25–26% in UTUC), RB1 loss (19–20% vs. 0–3%), and alterations in TERT, ERBB2, and CDKN1A, suggesting a cancer biology dominated by cell cycle dysregulation through disruption of the TP53/RB1 tumour suppressor axis rather than oncogenic kinase activation [5,12,13].

Furthermore, UTUC has a higher prevalence of microsatellite instability-high (MSI-H) and mismatch repair deficiency (dMMR) compared to bladder UC (3.2–7.2% vs. 1%), in line with the established association between UTUC and Lynch syndrome; UTUC represents the third most common Lynch-associated malignancy after colorectal and endometrial cancers, with germline pathogenic variants in MMR genes (MLH1, MSH2, MSH6, PMS2) trending higher in UTUC patients (1.8% vs. 0.6%), and almost half of patients harbouring MMR germline variants presenting with UTUC as their sentinel cancer, underscoring the importance of universal MMR/MSI testing in all UTUC cases for both therapeutic selection and cascade family screening [14,15].

The immune microenvironment also differs between the two anatomic sites: bladder UC demonstrates higher PD-L1 expression and is enriched for regulatory T cells and natural killer cells, whereas UTUC shows a predominance of CD4+ T cells and is strongly enriched for the luminal papillary (LumP) molecular subtype (comprising 57% of muscle-invasive UTUC vs. a lower proportion in bladder cancer), a transcriptomic classification characterised by FGFR3 pathway activation, low immune cell infiltration, and minimal PD-L1 expression, which may mechanistically explain the clinically observed pattern of increased treatment resistance to both platinum-based chemotherapy and immune checkpoint inhibitors in UTUC compared to bladder primary tumours [16,17].

Overall, these findings demonstrate that bladder UC and UTUC, despite similar histology and anatomic proximity, represent molecularly different diseases, requiring distinct biomarker-driven treatment approaches rather than a one-size-fits-all approach to urothelial carcinoma.

### 1.3. Literature Search Strategy

We conducted a narrative review of the published literature using PubMed/MEDLINE and Embase. The search included key terms related to upper tract urothelial carcinoma, diagnostic tests, and emerging biomarkers. More specifically, we searched with “upper tract urothelial cancer”, “UTUC,” “diagnosis,” “prognosis”, “biomarkers,” “DNA methylation,” “circulating tumour DNA,” and “FISH.” Priority was given to contemporary clinical guidelines, systematic reviews, and studies with clear relevance to clinical practice. Additional relevant articles were identified through manual search of reference lists of previously published articles. Our aim is to provide a clinically meaningful overview of current diagnostic approaches, their limitations and emerging tools that could manage these limitations.

## 2. Diagnosis and Risk Stratification of Upper Tract UC: Current Practice and Limitations

Diagnosis of upper tract urothelial carcinoma (UTUC) relies on a multimodal pathway incorporating cross-sectional imaging, voided or selective urinary cytology, and ureteroscopic visualisation with or without biopsy. While this approach achieves reasonable tumour detection, each modality suffers from limitations that affect sensitivity, specificity, grading/staging accuracy, and patient safety. It is imperative to perform cystoscopy as part of the diagnostic work up to exclude synchronous bladder cancer that can be found in up to 20% of the cases based on large retrospective series [18]. In the following section, we review the role and limitations of CT urography, urinary cytology, and diagnostic ureteroscopy with biopsy in the evaluation of UTUC. We also discuss the emerging role of adjunctive urinary and molecular biomarkers, in line with contemporary guideline recommendations and recent evidence.

### 2.1. Imaging Studies

CT urography (CTU) is the reference first-line imaging modality for suspected UTUC in most contemporary guidelines. A meta-analysis by Janisch et al., cited by both the EAU and AUA guidelines, reports excellent pooled sensitivity and specificity of approximately 92% and 95%, respectively, for detecting UTUC [19]. However, UTUC diagnostic accuracy seems to be limited by the size of the lesion in some studies. CTU cannot reliably detect flat lesions such us CIS or correctly classify the depth of invasion between non-invasive and microscopically invasive disease. Jinzaki et al. reported per segment sensitivity of 87% in 77 segments of confirmed upper tract UC, with false negative results associated with CIS or small papillary lesions < 10 mm [20]. In addition, false positives may arise from benign conditions such as inflammation, blood clots, stones or strictures. Sadow et al. reported a positive predictive value (PPV) of 53% in their series of 76 cases of suspected upper tract UC on CTU, with CTU performing significantly better for lesions > 5 mm (PPV 83%) compared to lesions < 5 mm (PPV 0%) or urothelial thickening (PPV 46%) [21]. CTU also entails exposure to ionising radiation and iodinated contrast which is problematic for patients with impaired renal function. This is important particularly in the context of nephron sparing surgery that requires frequent imaging follow up [22].

Imaging modalities that have been used historically in this setting, such us intravenous or retrograde uretero-pyelography and ultrasound of the urinary tract, have generally been replaced by CTU due to their lower sensitivity and specificity across different studies [20,23]. However, in certain populations with contraindication to cross-sectional imaging they remain a valid alternative with acceptable diagnostic accuracy [24].

MR urography is, also, an alternative when CTU is contraindicated (e.g., contrast allergy, radiation exposure). However, it has lower sensitivity comparing to CTU in detecting upper tract UC and remains less widely validated and available [25]. Recent studies suggest MRU can assist risk stratification of muscle invasion providing staging information, with reported sensitivity up to 95% and specificity ~71% for diagnosing muscle-layer invasion, albeit with variability across centres and protocols [26]. Finally, recent studies showed promising results of nuclear medicine modalities such as FDG-PET CT in staging lymph nodes and distant metastatic disease; however, further prospective studies are required to clarify their role in the diagnostic pathway of UTUC [27,28].

### 2.2. Ureteroscopy (URS) with Biopsy

Diagnostic URS enables direct visualisation, lesion mapping, and tissue diagnosis, and is integral to contemporary diagnostic pathways. It is still debatable if every single patient with suspected upper tract UC should undergo diagnostic URS and biopsy particularly if a positive high-grade cytology is confirmed. EAU guidelines recommend performing diagnostic URS if voided urine cytology and imaging are not sufficient for diagnosis or risk stratification, whereas AUA guidelines offer similar recommendations [8,9].

On a retrospective series of patients treated with radical nephroureterectomy based on CT diagnosis, 2.9% of patients were found to have benign disease. Voided urine cytology was negative and ureteroscopy was either not attempted or was technically not possible [29]. Similarly, data from the UK reported a rate up to 8% of benign histology among 863 patients who had nephroureterectomy for suspected upper tract UC [30]. Adding diagnostic URS on the diagnostic work up of suspected UTUC was found to decrease misdiagnoses from 15.5% to 2.1% in a study Tsivian et al., whereas it changed the treatment decision in over half of the patients in a series of 44 suspected upper tract UC published by Aumetell et al. [31,32].

On the other hand, diagnostic URS, particularly when accompanied by upper tract biopsy, has been associated with increased risk of bladder UC recurrence as demonstrated clearly in several meta-analyses [33,34]. In addition, a recent audit report from the UK reported that diagnostic URS was undertaken in approximately 60% of the patients eventually treated with nephroureterectomy and it appeared to delay radical treatment by 53 days in comparison to the patients treated with immediate nephroureterectomy. Interestingly, the diagnostic URS group was associated with a double fold increase in bladder recurrences, increased risk of positive surgical margins (for invasive stages) and possible worse long term oncological outcomes. Therefore, the authors proposed a risk-stratified approach in which positive urine cytology for high-grade cells and compelling imaging evidence of upper tract lesion would be sufficient to proceed with radical surgery without prior diagnostic URS [35]. Similar approach was recommended in another UK study by Veeratterapillay et al. They reported increased risk of bladder UC recurrence but no adverse impact on long term survival measures in patients who underwent diagnostic URS prior to nephroureterectomy in a single institution study with a long term follow up of almost 10 years [36].

Furthermore, ureteroscopic biopsy samples are often small and subject to crush artefacts, leading to under-grading and under-staging. Non diagnostic biopsies have been reported in up to 37% of cases in retrospective series, whereas staging information is usually limited due to insufficient biopsy depth. As reported in a review article by Gravestock et al., concordance in grade and stage between ureteroscopic biopsy and nephroureterectomy specimen have been variable across different studies ranging between 43 and 96% and 32–79%, respectively. Importantly, upgrading and upstaging rates exceeded 30% and 60%, respectively, in some studies, with significant implications on risk stratification, surgical management and prognosis [37].

In conclusion, the aggregate limitations of current tools including missed small/flat lesions on imaging and lack of discrimination between invasive and non-invasive disease on imaging, and imperfect grading/staging from URS biopsies can lead to both over- and under-treatment. These gaps provide the clinical rationale for integrating validated urinary and blood-based biomarkers alongside existing modalities in future diagnostic algorithms.

In current practice, the diagnostic evaluation of suspected UTUC typically follows a stepwise approach. Initial assessment is based on cross-sectional imaging with CT urography and cystoscopy, combined with voided or selective urinary cytology to identify high-risk disease. Diagnostic ureteroscopy with biopsy is generally reserved for cases with equivocal imaging or cytology, or when there is strong indication for kidney-sparing management such as in single kidney patients.

## 3. Diagnostic and Surveillance Liquid Biomarkers

**Figure 1:**
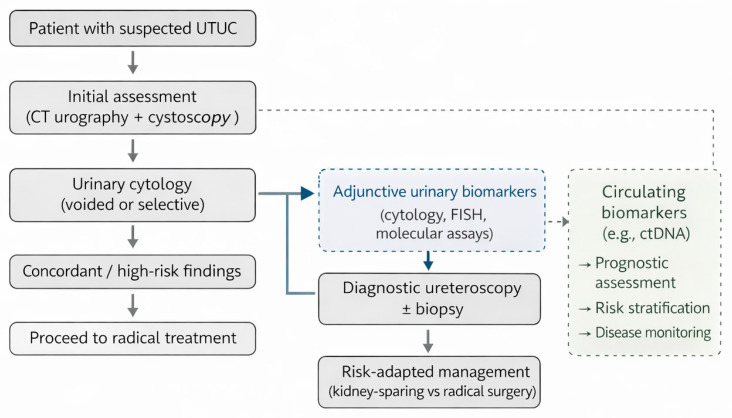
Summary of a proposed framework for integrating urinary and circulating biomarkers into the diagnostic pathway of upper tract urothelial carcinoma. While cross-sectional imaging, urinary cytology, and ureteroscopic assessment remain central to diagnosis, biomarkers may provide adjunctive diagnostic and risk-stratification information in selected clinical scenarios, particularly when standard investigations yield equivocal results. Circulating biomarkers, such as ctDNA, currently appear more suited to prognostic assessment and disease monitoring rather than primary tumour detection.

Beyond established diagnostic modalities, several emerging urinary and circulating biomarkers have been investigated as adjunctive tools for diagnosis, risk stratification, and surveillance (Figure 1). Recent reviews of molecular diagnostics in urothelial cancer have highlighted both the potential and the current limitations of liquid biopsy approaches. In an editorial overview, Leonardi et al. discussed advances in urinary and circulating biomarkers, including DNA methylation assays, and circulating tumour DNA, while emphasising key challenges related to assay standardisation, tumour heterogeneity, and clinical validation. Although much of the available evidence is derived from bladder cancer, the authors also noted emerging data supporting the applicability of selected molecular biomarkers in upper tract urothelial carcinoma, reinforcing the relevance of these approaches across the urothelial tract [38].

### 3.1. Urine Cytology

Urine cytology constitutes the commonest biomarker for detection of UTUC. It is an integral part of the initial assessment of suspected upper tract UC and has been included in the diagnostic algorithms of both EAU and AUA guidelines [8,9]. It has demonstrated high specificity—exceeding 90% across different studies—for high-grade (HG) disease [39]. A meta-analysis of selective cytology in UTUC (including upper tract catheterisation/aspiration, ureteral washings, and brush cytology) confirmed a high pooled specificity of 90% but only modest sensitivity of 53% across all histology grades, which improved to 69.9% for the high-grade histology group. However, there was significant heterogeneity across studies that limits the interpretation of the results [40]. Across other studies in the literature, urine cytology sensitivity ranges between 40% and 76% depending on tumour grade, stage, sampling method and reporting system [40,41,42,43]. In general, selective upper tract cytology is considered superior to voided cytology for upper tract UC due to higher cellular yield by avoiding dilution in the urine of the contralateral renal unit [44].

In a representative series of 144 cases of upper tract UC histologically confirmed following resection, Zhao et al. reported urine cytology (including a mixture of voided and selective upper tract cytology specimens) sensitivity of 64.7%, specificity of 64.3% and positive predictive value (PPV) of 93.8% for the diagnosis of high-grade disease. If atypical urine cytology was considered positive, then sensitivity increased to 92.2%. For the cases that had preoperative ureteroscopic biopsy, biopsy sensitivity for the diagnosis of upper tract UC was 59.2% and when biopsy and positive cytology were combined, sensitivity increased to 85.7% [43].

In general, voided urine cytology performs less well in UTUC compared to bladder UC due to lower cellular yield and dilution in voided samples. This limitation can markedly be mitigated by selective upper tract washings obtained via ureteral catheterisation or during ureteroscopy. It is pivotal to highlight that specimens should ideally be collected before contrast instillation, as prolonged exposure to ionic high-osmolar agents can degrade cellular morphology [45]. On a study with biopsy proven high-grade upper tract UC, Zhang et al. reported a sensitivity of 50% for preceding voided cytology as opposed to almost 90% for upper tract selective cytology washings [46].

In addition, the implementation of The Paris System for Reporting Urinary Cytology (TPS) has standardised terminology and reproducibility of urine cytology findings across various studies [47]. In contrast to the improvement of the diagnostic ability in bladder UC, the effect of TPS introduction in upper tract UC remains less clear. In general, variable rates of sensitivity have been reported across various retrospective studies with a consistently reported excellent specificity and positive predictive value (>90%) [42].

### 3.2. Urine DNA Aneuploidy and FISH

Fluorescent in situ hybridization (FISH) assays have long been used for diagnosing UC. More specifically, UroVysion^®^ FISH is the most validated molecular cytogenic assay that detects chromosomal aneuploidy (gains or deletions) in exfoliated cells in the urine involving centromeric enumeration probes (CEP) for chromosomes 3, 7, 17 and 9p21, which targets the CDKN2A/P16 tumour suppressor gene [48]. It got FDA approval in 2001 and has been widely tested in bladder UC for the assessment of haematuria, as a surveillance biomarker and as a prognostic biomarker after treatment including BCG immunotherapy. It has demonstrated satisfactory sensitivity and specificity but not sufficient to replace gold standard diagnostics such as cystoscopy [49].

In UTUC, FISH has demonstrated greater diagnostic sensitivity compared to urine cytology, across all histology groups and particularly for high-grade disease. However, most of the evidence comes from small number, single institution studies with heterogeneity in the way samples were collected, processed, and evaluated. Therefore, results need to be interpreted with caution. Similarly to urine cytology, FISH in selective upper tract samples has higher sensitivity for UTUC diagnosis compared to voided urine samples. Reassuringly, a small feasibility study suggested that selective upper tract samples in volumes as low as 1 mL can be sufficient for diagnosis [50,51].

In a meta-analysis of studies comparing FISH to cytology including more than 2000 patients in total, Jin et al. reported a pooled sensitivity of 84% and specificity of 89.5% for FISH comparing to 40% and 95.9% for voided urine cytology, respectively [52]. However, no breakdown of cases into high- and low risk UTUC was provided. A later meta-analysis by Aalami et al. including over 1000 patients, reported a pooled sensitivity of 72% and specificity of 95% for FISH in UTUC. Similarly, most of the studies included in this review were small, had variable control groups and the meta-analysis provided no breakdown between selective or voided urine cytology and between high- and low-grade disease [53]. On the largest individual retrospective studies, sensitivity of FISH seems to outperform urine cytology and range between 52% and 100% at the cost of more false positive results. In addition, the negative predictive value (NPV) of FISH across different studies varies between 50% and 100%, which makes it unreliable as a sole test to exclude cancer in a diagnostic or surveillance setting [52].

In addition, evidence from retrospective studies suggests the potential value of FISH as predictor of disease aggressiveness and as a risk factor for bladder recurrence following treatment. Su et al. demonstrated that positive FISH in voided urine was associated with higher tumour stage and grade in their series of 210 patients treated with radical surgery [54]. In another report from the same centre, Xu et al. included UTUC with low-risk preoperative features (negative cytology, non-invasive features on imaging, no hydronephrosis) and demonstrated that positive FISH was significantly associated with high-grade histology and advanced tumour stage (≥T2) at radical nephroureterectomy specimens [55]. Finally, Eismman et al. demonstrated that introduction of FISH in cases of ambiguous cytology or imaging allowed diagnosis of organ confined UTUC at earlier stages comparing to their cohort prior to the introduction of FISH [56]. In conclusion, the wide variations in reported characteristics would not allow FISH to safely replace current gold standard imaging and invasive modalities in the diagnosis and surveillance of UTUC but it may have a role as an adjunct particularly in cases of atypical or suspicious cytology results or ureteroscopy and biopsy are not possible or inconclusive [8].

### 3.3. Urine Genetic and Transcriptomic Biomarkers

Urine cell-free DNA has been tested for detection of somatic hotspot mutations in different tumour suppressor or oncogenes such as TERT (telomerase reverse transcriptase), FGFR3 (fibroblast growth factor receptor 3), TP53 (tumour protein 53) and PIK3CA (phosphatidylinositol-4,5-biphosphate 3-kinase catalytic sub-unit alpha), in patients with UTUC. Despite the quite encouraging results, evidence is limited to small studies and further external validation is required before wider implementation.

Hayashi et al. assessed a cohort of 153 patients for TERT promoter and FGFR3 hotspot mutations in urine cell-free DNA, including 56 patients with localised UTUC, 50 patients with haematuria of different causes, 21 patients with previous UC and 26 healthy patients. They reported a significantly increased amount of extracted cell-free DNA in the UTUC cohort comparing to the other cohorts but no correlation between the amount of cell-free DNA and grade or stage of disease was found. In addition, TERT promoter (C228T and C250T) and FGFR3 hotspot mutations were significantly more common in the UTUC cohort comparing to controls and demonstrated a sensitivity and specificity of 55.4% and 100%, respectively, for UTUC diagnosis when analysed against the control cohort. Combination of the hotspot mutations and cytology further improved sensitivity to 78.6% whilst maintaining an excellent specificity of 96% [57].

In addition, Ouyang et al. combined TERT and FGFR3 mutation analysis with NRN2 (neuronin 1) methylation profiling in hybrid urine assay. In their prospective study of 95 patients with UTUC against a control arm of 307 patients, the combined assay panel achieved a sensitivity of 91.6% and specificity of 94.8% (AUC 0.958, 95% CI: 0.933–0.975), substantially outperforming either mutation or methylation markers alone. The incorporation of patient’s age as a clinical covariate, further improved diagnostic rates with sensitivity 93.7% and specificity of 94.4% [58]. Extending these findings, Wei et al. evaluated a combined cell-free DNA DNA-methylation + 17-gene mutation panel in 45 patients with suspected UTUC. The most frequent mutations were TERT (51.4%) and TP53 (25.7%), followed by HRAS (20%), PIK3CA (11.4%) and KRAS (5.7%). Compared with cytology—29% sensitivity and 100% specificity—the molecular panel achieved 86% sensitivity, 80% specificity, and AUC 0.829 (*p* < 0.001), representing a significant improvement (ΔAUC = 0.186, *p* = 0.023) and demonstrated a moderate agreement with tissue pathology (κ = 0.59, *p* < 0.001). These results further substantiate the diagnostic value of integrating mutation and methylation markers within the same cell-free DNA workflow [59].

Emerging RNA-based assays, which have widely been studied in detection of bladder cancer, could further contribute to molecular diagnosis of UTUC. The Xpert^®^ BC assay have been assessed in two prospective studies for UTUC. It is based on the detection of five different target mRNAs-ABL1, CRH, IGF2, UPK1B, and ANXA10—within a fully automated, cartridge-based RT-PCR system. In the study by D’ Elia et al., 82 patients underwent 111 diagnostic ureteroscopies and upper tract selective samples were analysed with Xpert^®^ BC-Detection, cytology, or FISH Urovysion^®^. Diagnostic performance of each modality was assessed against retrograde studies, ureteroscopy and/or biopsy. Overall sensitivity was 100% for Xpert^®^ BC-Detection, 51.9% for cytology, and 92.6% for Urovysion^®^-FISH test. However, specificity was 16.7% for Xpert^®^ BC-Detection, 95% for cytology, and 85% for Urovysion^®^-FISH [60]. Similar results were obtained in a subsequent study by the same group, in which Xpert^®^ BC-Detection demonstrated again sensitivity of 100% but specificity of only 4.5%. These results agree with previous studies on bladder UC. Although the high sensitivity could make Xpert^®^ BC-Detection an excellent rule out test, its poor specificity will inevitably lead to a high number of false positive results and thus, would not significantly reduce the number of invasive procedures such as diagnostic ureteroscopy [60,61].

### 3.4. Urine DNA Methylation Biomarkers

Another epigenetic event, occurring early in the urothelial carcinogenesis, is aberrant DNA methylation. These alterations can be detected in exfoliated urothelial cells and urinary cell-free DNA, constituting a robust biologically substrate for non-invasive UTUC diagnosis. Montero-Reis et al. investigated promoter methylation of three different genes (VIM, TMEFF2 and GDF15) in urine from UTUC and healthy controls. The combination of three epigenetic markers in one panel, identified sensitivity of 91% and specificity of 100%, while low VIM promoter levels, independently prognosticated poorer disease-specific survival [62]. Extending this concept to a wider 10-gene panel (ABCC6, BRCA1, CDH1, GDF15, HSPA2, RASSF1A, SALL3, THBS1, TMEFF2, VIM), among which CDH1, GDF15, HSPA2, RASSF1A, TMEFF2, and VIM independently predicted UTUC, demonstrating sensitivity of 82% and specificity of 68% [63]. ONECUT2, another methylation biomarker introduced by Xu et al., also demonstrated high sensitivity and negative predictive value (NPV—94.7%) as part of a multi-analyte assay with 17 mutation targets [64]. AUC of 0.879 (*p* < 0.001) and balanced diagnostic accuracy for the same methylation loci was also exhibited by Wei et al. who included it in their cell-free DNA-based workflows [65]. Fujimoto et al. corroborated these findings in tumour tissues, correlating methylation burden to increasing stage and grade of the disease, thereby linking these epigenetic events to malignant progression. Additionally, Fujimoto et al. in their tissue-based study, using genome-wide methylation arrays, identified 2448 UTUC-specific CpG methylation sites. Sensitivity of 86.66–100% and specificity 93.5–100% (AUC 0.959–1) was demonstrated with ten loci panel, including BARHL2, FMN2, RADIL and LOC154822 among others. A four-locus assay, combining these markers, exhibited 97.5% sensitivity and 100% specificity in identification of UTUC. These numbers were independent of grade or stage, highlighting their efficacy in early-stage cancer detection and surveillance utilisation [66].

In terms of commercially available methylation assays, multiple studies have described their role in bladder cancer detection. The Bladder EpiCheck^®^ test has also demonstrated clinical value in diagnosis of UTUC. Including 15 methylation sites, it had consistently outperformed urine cytology. As per Ricciardi et al. (2025), across five cohorts, sensitivity ranged from 64.5% to 97.4%, specificity from 78.8% to 100% and negative predictive value from 73% to 97%, particularly in HG UTUC [67]. Characteristically, a meta-analysis of 334 patients yielded a pooled sensitivity of 85%, specificity of 93%, positive predictive value (PPV) of 74% and NPV of 84% [68]. Similarly, the Bladder CARE^®^ assay, based on methylation of tRNA-Cys, SIM2, and NKX1-1, demonstrated 96% sensitivity, 88% specificity, and AUC > 0.9, significantly outperforming cytology [69].

A metanalysis of nine studies encompassing 1326 patients evaluating different urine DNA-methylation assays (including VIM, TMEFF2, GDF15, NRN1, ONECUT2, and the Bladder EpiCheck^®^ panel), demonstrated pooled sensitivity of 89% (95%CI 83–93%) and specificity of 91% (95%CI 0.83–0.96). The overall AUC was identified 0.96 (95%CI 0.93–0.97), while the subgroup and meta-regression analyses demonstrated no significant differences on the results related to urine-collection method, analytic platform or tumour grade, highlighting the reproducibility of methylation-based diagnostics [70]. Lastly, emerging methylation markers such as ITIH5 and ECRG4 as described by Rose et al., have shown promising diagnostic value (sensitivity 64.3%, specificity 81.5%) in urothelial/bladder cancer urine assays, and may enhance multiplex panels when combined with established platforms such as EpiCheck^®^ in detecting UTUC [71].

### 3.5. Urine Protein Biomarkers

Urinary protein biomarkers have also been developed to enhance non-invasive diagnosis and surveillance of urothelial malignancies. They have been assessed mainly in cohorts with bladder UC; however, the biological rationale, urine-based methodology and relative low cost, might make it a plausible tool for non-invasive detection and monitoring of UTUC [72]. Jovanovic et al. assessed the diagnostic value of the Nuclear Matrix Protein 22 (NMP22) urine test in 34 patients with upper tract UC and 25 controls in voided urine and selective upper tract samples. They reported sensitivity > 70% and specificity approximately 90% that were comparable between voided and selective upper tract samples and superior to equivalent urine cytology samples [73]. Similarly, Wong et al. assessed the urine protein test BTA, which detects complement factor H-related proteins in urine, in combination with other biomarkers including urine cytology and Survivin, and reported sensitivity 86% and specificity 96% in a cohort of 44 patients with upper tract UC [74]. Despite the promising results, larger studies are required to establish the role of protein biomarkers in upper tract UC.

### 3.6. Serum Biomarkers

Some serum biomarkers have shown very promising potential in UC. Most of the studies reported to date include patients with UC of the bladder and demonstrated diagnostic and prognostic potential as well as improved staging and detection of minimal residual disease post radical treatment. Circulating tumour DNA (ctDNA) is an innovative UC marker that has been assessed in well-designed randomised controlled trials (RCT) that included patients with bladder and upper tract UC [75]. It involves assessing cell-free tumour DNA released by cancer cells to the circulation utilising either tumour informed or tumour agnostic approaches [76].

In a prospective single cohort study, Huelster et al. explored the potential of ctDNA as a predictive biomarker for muscle invasive/locally advanced disease in a cohort of 30 upper tract UC histologically confirmed following extirpative surgery. The authors, using a threshold of two molecular alternations for ctDNA positivity, reported a 71% sensitivity and 94% specificity to predict muscle invasive/locally advanced disease. Combining this threshold with a plasma copy number burden score threshold > 6.5 marginally increased sensitivity to 79%. In addition, positive ctDNA status before extirpative surgery was strongly associated with disease progression and mortality at a median follow up of 14 months [77]. In addition, Nakano et al. reported that ctDNA positivity (defined as >2% fraction of cell-free DNA) increased with histopathological depth in their series of 50 patients with upper tract UC. Similarly, to the previous study, ctDNA positivity and following radical surgery was associated with worse survival outcomes [78]. These prognostic properties of ctDNA agree with the results of large well-designed RCTs on bladder UC following radical treatment [79].

Lastly, similarly to the paradigm of bladder UC, ctDNA post radical treatment might inform on the need of postoperative immunotherapy in patients stratified as high risk of recurrence based on the ctDNA status [80]. In a recent abstract, Huang et al. presented the results of the CURATE-UTUC study (NCT05595408), which is a multicentre prospective longitudinal cohort study that explored the role of ctDNA and urinary tumour DNA (utDNA) dynamics for predicting minimal residual disease and recurrence risk in invasive and locally advanced upper tract urothelial carcinoma (≥pT2/N+). All 78 participants had radical nephroureterectomy and were planned to start adjuvant treatment within 3 months. The authors reported that ctDNA/utDNA predicted disease recurrence in 94% of the patients who eventually relapsed in a median follow up of 15 months. Importantly, liquid biopsy positive result preceded clinical recurrence by a median of 106 days. ctDNA positivity post-surgery generally predicted extravesical recurrence and utDNA positivity intravesical recurrence. The full report of this study is awaited but results appear consistent with the ctDNA trials in bladder cancer and might change the landscape in disease monitoring, choice and timing of adjuvant treatment with direct survival benefit [81]. Overall, ctDNA represents a significant advancement in UC management that is likely to alter the risk stratification, treatment options and disease monitoring; however, larger validating studies are required to fully elicit their utility in UTUC.

Finally, small non-coding RNAs have also been evaluated for their diagnostic efficiency in UTUC. These studies are still small and the results preliminary but they might inform larger validating studies to fully explore the potential of small non-coding RNAs. Kriebel et al. assessed the expression of 11 miRNAs in serum and upper tract UC tissue samples in 47 patients with upper tract UC confirmed with nephroureterectomy and reported that 8 of the 11 miRNAs expression was upregulated in cancer tissue samples comparing to controls. Also, serum miRNA-141 levels were significantly higher in the serum samples of patients with upper tract UC comparing to controls with a reported sensitivity and specificity in predicting upper tract UC at 71% and 74%, respectively [82]. Similarly, Tao et al. reported increased serum concentration of 13 miRNAs in 46 patients with upper tract UC in comparison to 30 cancer-free controls with haematuria with reported sensitivity ranging between 59% and 98% and specificity between 70% and 100%. Ten of them presenting an AUC > 0.8 for tumour detection with the most effective ones being the miR-664a-3p, miR-431-5p, and let-7c [83].

Table 1 summarises the diagnostic performance of key diagnostic tests and biomarkers for UTUC. Sensitivity and specificity are shown as ranges to reflect differences between studies.

## 4. Conclusions

Upper tract UC is a rare disease and as such large prospective trials are often lacking to support evidence based-decisions. Diagnostic modalities and risk stratification remains far from perfect risking under- or overtreatment in a significant proportion of patients. Although contemporary CTU scans have a high sensitivity to pick up upper tract lesions exceeding 90%, they cannot reliably differentiate between invasive and non-invasive disease and are likely to miss flat or small lesions [8,9]. Urine cytology with specificity and PPV reliably exceeding 90% across different studies can act as an adjunct to diagnose high-grade disease. However, urine cytology suffers from low sensitivity and thus, leaves a significant proportion of high-risk tumours and most of the low-risk tumours undiagnosed. FISH technologies could be useful as an adjunct particularly in the setting of non-diagnostic/atypical cytology. However, due to their variable diagnostic ability, they cannot be reliably used as sole non-invasive diagnostic replacing invasive ureteroscopy. Emerging urinary and circulating biomarkers, including DNA mutation and methylation assays and circulating tumour DNA, demonstrate promising diagnostic and prognostic performance. These approaches may enhance preoperative risk stratification, reduce the need for invasive diagnostics, and improve decision making between kidney-sparing and radical surgical strategies. However, most biomarkers remain investigational and require validation in large, prospective, multi-centre studies before routine clinical implementation.

A future strategy addressing upper tract urothelial carcinoma (UTUC) may integrate advanced imaging with liquid biopsy techniques to enable non-invasive determination of tumour grade and stage. In appropriately selected patients, neoadjuvant systemic therapy could be administered, followed by repeat liquid biopsy to assess response, further refine risk stratification and guide the choice between nephron-sparing operations and radical surgery. Biomarker-based surveillance strategies may be implemented to monitor disease progression or recurrence post conservative treatments, thereby reducing the need for invasive procedures while enhancing the overall precision of diagnostic and management pathways.

In conclusion, future research should also prioritise standardisation of urinary biomarker reporting, including assay thresholds, sampling methods, and outcome definitions, to improve comparability across studies. In parallel, advances in artificial intelligence–assisted image analysis and multivariable risk prediction tools may further enhance diagnostic accuracy, personalised risk stratification and cost effectiveness in UTUC.

## Figures and Tables

**Table 1:** Summary of diagnostic performance of key diagnostic modalities and biomarkers UTUC ^a^.

Diagnostic Modality/Biomarker	Sample	Sensitivity (%)	Specificity (%)	Key Strengths	Key Limitations
CT urography (CTU) [19,20]	Imaging	87–92	~95	First-line imaging; high diagnostic accuracy	Limited for CIS/small lesions; radiation/contrast exposure
Urine cytology (UTUC overall) [39,40,41,42,43]	Urine	40–76	>90	High specificity (esp. high-grade)	Limited sensitivity; depends on sampling/reporting
Urine cytology (selective, high-grade) [39,40]	Urine	~69.9	~90	Best cytology performance in HG disease	Still misses a substantial fraction of HG tumours
UroVysion^®^ FISH [52]	Urine	84	89.5	Higher sensitivity than cytology in many series	False positives; NPV variable
^b^ Urine DNA mutation panels [57,58]	Urine cell-free DNA	55.4–91.6	94.8–100	Very high specificity; improves with multi-analyte panels	Variable performance; validation needed
^b^ Urine DNA methylation assays [62,63,64,65,66,67,68,70]	Urine	64.5–100	68–100	High diagnostic accuracy in multiple panels	Platform heterogeneity; standardisation/cost issues
RNA-based assays (Xpert^®^ BC) [60,61]	Urine	100	4.5–16.7	Excellent sensitivity	Very poor specificity
^c^ Urine protein biomarkers (e.g., NMP22; BTA-based combinations) [73,74]	Urine	70–86	90–96	Potentially low-cost; non-invasive	Very limited UTUC cohorts; heterogeneous assays; not validated
^c^ Serum microRNAs (miRNA) [82,83]	Serum	59–98	70–100	Minimally invasive; potential diagnostic signature	Exploratory; heterogeneity; limited validation
^d^ Circulating tumour DNA (ctDNA) [77,78,79]	Plasma	71–79	~94	Risk stratification; prognostic/MRD potential	Not a primary screening tool; limited UTUC cohorts

^a^ Reported sensitivity and specificity ranges reflect values derived primarily from meta-analyses and larger prospective studies cited in the text, rather than absolute extremes from small heterogeneous cohorts. ^b^ Several different urine mutation and methylation panels have been described. Each panel has its own diagnostic performance. The sensitivity and specificity shown in the table represent the range across these panels. ^c^ Urinary protein and serum microRNA biomarkers demonstrated diagnostic potential in exploratory studies; however, with limited cohort sizes, heterogeneity, and absence of validation studies. ^d^ Circulating tumour DNA performance metrics relate primarily to prediction of muscle-invasive or locally advanced disease and detection of MRD rather than primary tumour screening.

## Data Availability

No new data were created or analyzed in this study. Data sharing is not applicable to this article.
